# Bacterial 16S Ribosomal Gene Fingerprints as a Tool to Diagnose and Mitigate Fish Larvae Gut Dysbiosis

**DOI:** 10.1111/1758-2229.70187

**Published:** 2025-10-03

**Authors:** Babak Najafpour, Adelino V. M. Canario, Deborah M. Power

**Affiliations:** ^1^ CCMAR/CIMAR Algarve Centre of Marine Sciences Universidade do Algarve Faro Portugal; ^2^ Department of Biological Sciences University of Calgary, University Drive Calgary Canada; ^3^ International Research Center for Marine Biosciences, Ministry of Science and Technology Shanghai Ocean University Shanghai China

**Keywords:** bacterial diversity, dysbiosis, larval performance, mediterranean hatcheries, pathogens

## Abstract

Dysbiosis is associated with shifts in the diversity or relative abundance of beneficial versus harmful bacteria, leading to health issues in organisms. This study investigated gut bacterial dysbiosis associated with larval quality using 16S rRNA gene sequencing. The gut microbiome of gilthead sea bream and European sea bass, key commercial species and vertebrate models, was examined in high‐ and low‐quality larvae batches from several European hatcheries. Larval quality, hatchery site and species influenced bacterial diversity in the gut. Individuals from larval batches that performed well had higher microbial diversity in the gut and individuals from batches that performed poorly had a gut microbiota dominated by pathogenic *Vibrio* (e.g., *V. aestuarianus* and *V. cortegadensis*). The bacterial dysbiosis index revealed a notable predominance of Fusobacteriota and Firmicutes phyla, *Thermoanaerobacteria* class and *Lactobacillaceae*, *Moritellaceae*, *Clostridiaceae*, *Thiotrichaceae* and *Shewanellaceae* families in good‐quality larvae batches, and a prevalence of the Proteobacteria phylum, *Gammaproteobacteria* class, *Sphingomonadaceae* and *Vibrionaceae* families in the gut of individuals from poor‐quality larvae batches. A positive dysbiosis index (cutoff > 0.4) was associated with a high risk of decreased larval performance and quality. Additionally, the abundance of *Clostridium_sensu_stricto_15*, *Shewanellaceae_unclassified*, *Cetobacterium*, *Psychrilyobacter*, *Moritella* and *Latilactobacillus* genera in the gut of good production batches, and the *Vibrio* genus in the gut of poor production batches, identified these genus as potential markers for diagnosing and mitigating bacterial dysbiosis in fish and potentially other vertebrates.

## Graphical abstract

This study profiles the gut microbiota of poor‐ and good‐quality seabream and seabass larvae across different sites using 16S rRNA sequencing. Several bacterial markers associated with larval quality were identified and used to develop a dysbiosis index. Gut bacterial correlation networks revealed potential bacteria–bacteria interactions. These tools are applicable for diagnosing and mitigating gut dysbiosis in marine fish under a broad range of conditions and, potentially, in other vertebrates with similar bacterial profiles.

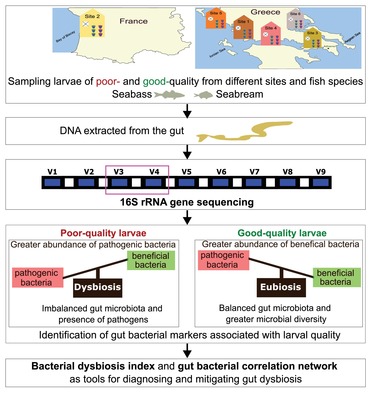

## 1 Introduction

The potential diversity of symbiotic relationships between microorganisms and their animal host, the holobiont, is vast (Rosenberg and Zilber‐Rosenberg 2011). Microbiota can influence the host's nutrition, protect it against pathogens and modulate its immune response and development (Rosenberg and Zilber‐Rosenberg 2018). The importance for host physiology of the microbiota is highlighted by, for example, the role of gut microbes in promoting intestinal angiogenesis in humans (Franks 2013). However, functional contributions from the microbiota and their products to the host remain largely unknown. With the advent of metagenomic approaches, the limitations associated with culture‐based methods for investigating the microbiome, such as the influence of culture media on bacterial abundance and diversity, have been largely overcome (Spanggaard et al. 2000). The emergence of amplicon‐based techniques targeting taxonomic marker genes, such as *16S ribosomal RNA* (rRNA), alongside metatranscriptomics, is providing a comprehensive understanding of microbial composition and functionality within diverse ecological niches (Simon and Daniel 2011; Aßhauer et al. 2015).

The gut hosts the largest microbial population compared to other organs. Consequently, extensive research in mammals and humans has prioritised studies of how the gut microbiome influences the host (de Jonge et al. 2022; Thursby and Juge 2017). However, studies of fish gut microbiota are far more limited (reviewed by Egerton et al. 2018). Given the exponential and continuing development of aquaculture and the demand for rapid production of high‐quality fish, there is a growing interest in the influence of the microbiota on production traits and the management of the gut microbiome (Egerton et al. 2018). The manipulation of the gut microbiota with probiotics and prebiotics is proposed as a sustainable means to promote resistance to pathogens, stimulate growth, refine lipid metabolism and enhance the immune response in fish (Tellez et al. 2006; Newaj‐Fyzul et al. 2014; Ringø et al. 2014). In mammals, various factors have been identified that influence the gut microbiota, including host genetics, diet, age, birth and antibiotics (Hasan and Yang 2019). In fish, the rearing environment emerges as a pivotal factor that influences the gut microbiota, as shown by studies of the developing gut microbiota in Nile tilapia, *Oreochromis niloticus* , and grey mullet, *Mugil cephalus* (Giatsis et al. 2015; Le and Wang 2020). Changes in water physiochemical parameters can modify the aquatic microbial community as well as the fish gut microbiota and can vary with species and genotypes (Li et al. 2014; Carla Piazzon et al. 2020; Schmautz et al. 2021). Given the vast diversity of fish species and the many potential factors that can shape their gut microbiota, significant knowledge gaps remain about how its microbial composition influences performance (Egerton et al. 2018).

The European sea bass ( *Dicentrarchus labrax* ) and the gilthead sea bream ( *Sparus aurata* ) are major aquaculture species in the Mediterranean basin. Nonetheless, high mortalities in early developmental stages and unpredictable larval quality remain a bottleneck for their sustainable production (Llewellyn et al. 2014; Muniesa et al. 2020). Pathogens such as *Vibrio alginolyticus* , *V. parahemolyticus* and *Photobacterium damselae* subsp. *damselae* are associated with mass mortalities as a result of suboptimal water quality, host susceptibility, age‐related vulnerabilities and the virulence of pathogens (Abdel‐Aziz et al. 2013). Most studies of the gut microbiome in European sea bass and gilthead sea bream have been performed on post‐larva and adults, with limited attention given to larval stages and with few results from fish in a production setting (Carda‐Diéguez et al. 2014; Estruch et al. 2015; Nikouli et al. 2018; Serra et al. 2021). In addition, the literature provides only limited evidence linking animal performance and productivity to alterations in microbiome diversity and structure (Infante‐Villamil et al. 2021).

Larvae microbiomes from commercial hatcheries identified the core and common bacterial genera in standard quality production batches, with evidence suggesting that bacterial abundance and larval quality varied with hatchery (Califano et al. 2017). The present study was designed to determine the potential association between the larval gastrointestinal tract (gut) microbiome of commercially produced gilthead sea bream and European sea bass, their hatchery of origin and their performance (good or bad) scored by hatchery managers. The ultimate goal was to determine the potential link between larval quality and gut bacterial profiles to leverage a realistic approach for analysis of gut dysbiosis in larval stages of marine fish, particularly gilthead sea bream and European sea bass. We found bacterial communities associated with the quality of larval production batches and suggest some members of these communities can be used as potential predictors of gut health. We propose this approach as a reliable method for analysing gut dysbiosis in marine fish.

## 2 Materials and Methods

### 2.1 Experimental Design

**FIGURE 1:**
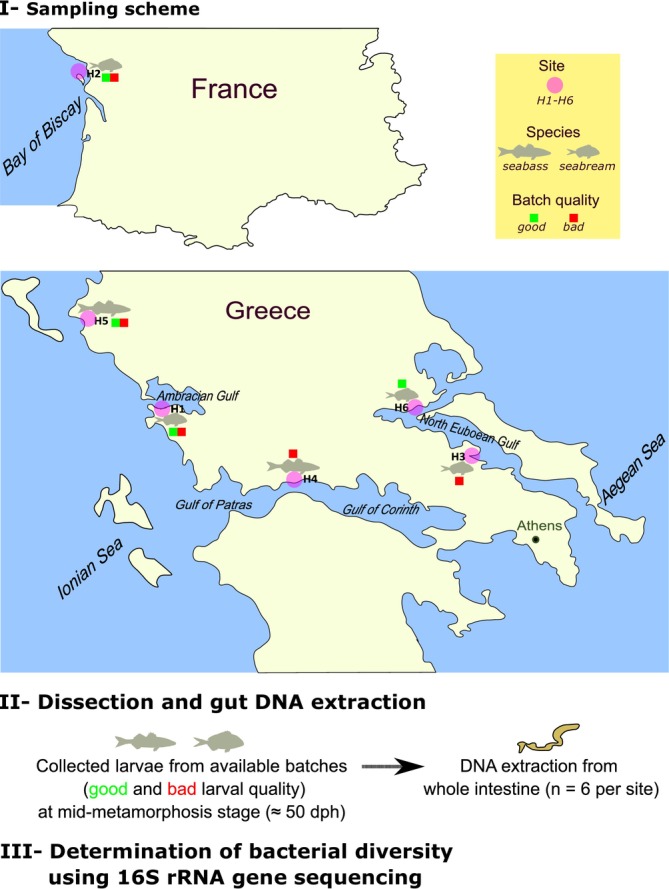
An overview of the experimental design for the analysis of the whole gut bacteria profiles of hatchery produced European sea bass and gilthead sea bream larvae. Samples of good and poor quality larvae (≈50 dph) from different production batches were collected from six hatcheries (H1–H6) in Greece and France and were fixed in RNAlater until analysis.

European sea bass and gilthead sea bream larvae approximately 50 days post‐hatch were obtained from standard production cycles of six hatcheries in Greece and France (Figure 1) and stored in RNA later (1:10 v/v) at −20°C until dissection and DNA extraction for production details and larval stages see Kourkouta et al. (2022) and Najafpour et al. (2024). The hatchery managers classified larval batches from production cycles as ‘good’ or ‘bad’ quality using criteria such as global malformation incidence, survival and growth rate. Larvae batch weights (Table S1) and skeletal abnormalities were the most important key‐performance‐indicators (KPIs) used in the collaborating finfish hatcheries (Kourkouta et al. 2022).

### 2.2 DNA Extraction and Sequencing

Whole larvae intestines of individuals from the ‘best’ and ‘poorest’ production batches (*n* = 3 larvae per batch) were dissected out on ice using a microscope with a cold light source. The gut DNA was extracted using a DNeasy Blood & Tissue Kit (Qiagen, Germany) optimised for fish larvae (Najafpour et al. 2023). Each of the collected guts was placed in 400 μL of lysis buffer and mechanically disrupted, initially with two iron beads per tube (Qiagen stainless steel beads of 5 mm) and three cycles of 30 s at 30 Hz in a Tissue Lyser (Qiagen). After the iron beads were removed, 400 mg of 0.1 mm zirconia/silica beads were added and the tissue was further disrupted in three cycles of 5 min at 25 Hz.

The DNA was shipped on ice to Stab Vida, Lda (Lisbon, Portugal) for sequencing. After checking the quality of the extracted DNA using a Qubit 2 fluorometer (ThermoFisher Scientific, Lisbon, Portugal) and 1% agarose gel electrophoresis, only samples with an absorption ratio > 1.8 and > 30 ng/μL yield were selected for library preparation and sequencing. Thirty‐two 16S rRNA gene libraries were constructed using an Illumina 16S Metagenomic Sequencing Library preparation protocol, with 12.5 ng DNA per sample and primers targeting the V3 and V4 hypervariable regions of the 16S rRNA gene for amplification (Klindworth et al. 2013). For sequencing, the MiSeq Reagent Kit v3 was used and generated 300 bp paired‐end sequencing reads in an Illumina MiSeq instrument.

### 2.3 Sequence Processing and Bioinformatics

The raw reads underwent processing and analysis through two distinct pipelines: the QIIME 2 pipeline (Bolyen et al. 2019), used by Stab Vida Lda (Lisbon, Portugal), as detailed in Najafpour et al. (2021), and the MiSeq_SOP pipeline using Mothur v.1.48.0 (Schloss et al. 2009; Kozich et al. 2013). Based on the sequencing statistics, the results obtained using the two pipelines were closely aligned (Table S2).

With the MiSeq_SOP pipeline, an additional filtering step was used to eliminate undesirable reads from chloroplasts, mitochondria, archaea, eukaryotes or of unknown origin. Subsequently, the phylotype command was employed to assign sequences to OTUs based on their taxonomic classification, using the SILVA database (silva.nr.v138_1) as a reference for mapping and taxonomy assignment. The obtained OTU table was used for the downstream analysis. An Amplicon Sequence Variant (ASV) approach (Schloss 2021) was implemented to cluster sequences based on their similarity (sequences were only allowed to differ by one or two bases). The sequencing output was used to develop qPCR primers targeting potentially abundant pathogens. The topmost abundant bacteria were specified, and diversity indices were calculated and visualised using microeco v. 0.17.0 (Liu et al. 2021).

The phylogenetic distance between the microbial communities in the samples was determined using the plot_ordination function and Bray–Curtis dissimilarity in phyloseq v. 1.38.0 (McMurdie and Holmes 2013). For this analysis, several ordination techniques were used to reduce method‐specific biases and effectively discriminate differences that were best captured by the model with the best fit to the data: Principal Component Analysis (PCA), Correspondence Analysis (CA), Principal Coordinate Analysis (PCoA), Nonmetric Multidimensional Scaling (NMDS) and Redundancy Analysis (RDA). The R packages ggplot2 v. 3.4.1 and cowplot v. 1.1.1 were used to visualise and merge plots.

The OTUs primarily contributing to beta diversity (encompassing the most abundant and most highly variable OTUs) were specified at the genus level using the simper function within vegan v. 2.6‐5 (Oksanen et al. 2020). The simper (similarity percentages) method generated comprehensive pairwise comparisons for categorical variables, including larval quality (good/bad), site and species (seabream/seabass). To predict the functional profiles of microbial communities, the data were mined using R microeco v1.8 and Tax4Fun2 v. 1.1.5 packages (Wemheuer et al. 2020; Liu et al. 2021). The functional analysis predicted OTU percentages for each trait within a network module. Changes in alpha and beta diversity in relation to larval quality, species and hatchery were analysed using several statistical methods in the microeco package.

### 2.4 Statistical Analysis

To mitigate the impact of sequencing depth on diversity measurements, the sequencing data were rarefied to the smallest total number of sequences across samples. Data normality was assessed using the Shapiro–Wilk normality test based on different variables, including larval quality (‘good’ vs. ‘bad’ conditions), species (sea bream and sea bass) and hatchery (six different hatcheries). The most appropriate statistical tests were applied based on the data distribution, and included Kruskal–Wallis, Wilcoxon and ANOVA. The Kruskal–Wallis Rank Sum Test was used to compare three or more independent groups, and the Wilcoxon Rank Sum Test was used to compare two independent groups, and they were applied to analyse statistical differences in diversity indices based on the variables quality, species and hatchery. The diversity indices based on the variable hatchery were further analysed using ANOVA. Diversity indices underwent further analysis using a generalised linear mixed‐effects model (GLMM) with a maximum likelihood fit employing the *glmer* function within lme4 v. 1.1‐32 (Bates et al. 2015). This model incorporated larval quality as fixed effects and hatchery and species as random effects. The best linear unbiased prediction (BLUP) was used to estimate random effects using the *ranef* function.

Beta diversity was compared using permutational analysis of variance (PERMANOVA) through the *cal_manova* function in microeco, developed based on the *adonis2* function and Bray–Curtis distance in vegan. The *filter_taxa* function in microeco retained 273 features with an abundance threshold higher than 0.0001 and an occurrence frequency threshold higher than 0.1 (features present in at least 10% of the samples). MaAsLin2 v. 1.8.0 was used to identify multivariable associations between microbial features and experimental metadata (Mallick et al. 2021). A general linear model was employed, incorporating fixed effects for quality and random effects for hatchery and species. Moreover, the bacteria profiles of good and bad‐quality larvae were compared using the web‐based platform MicrobiomeAnalyst (Chong et al. 2020), incorporating the statistical methods EdgeR and DESeq2, to identify significant features and potential biomarkers.

Bacterial genera found to be significantly different between good and bad larvae batches through at least one of the statistical methods used (GLM, EdgeR and DESeq2) were visualised in heatmaps using pheatmap v. 1.0.12 (Barter and Yu 2021). Our integrated approach employed several statistical methods to analyse the data, and took advantage of the strengths and limitations of each approach, including the potential influence of factors such as sample size, data distribution, variance structure and feature abundance on their outputs. For instance, some methods handle small sample sizes better, while others are more sensitive to low‐abundance features, and vice versa. Additionally, the normalisation approaches and statistical models used by each method differ significantly, meaning they do not deliver identical outputs. The approach used provided robust cross‐validation since we prioritised taxa that were consistently identified as significantly different (*p* < 0.05) with all approaches used. The approach also captured features that might have been top‐ranking in some methods but masked by others. CCREPE (Compositionality Corrected by REnormalization and PErmutation) v. 1.40.0, which uses the NC‐score, a similarity measure, to detect association patterns in microbial communities was used for analyses (Gevers et al. 2014). Two types of analyses were performed using CCREPE and Spearman correlation to identify correlation networks. The first analysis was performed using a single input of bacterial abundance, including gut samples from larvae classified as good or bad. In the second analysis, two separate inputs of bacterial abundance were used: one for the gut of good‐quality larvae and one for the gut of bad‐quality larvae.

### 2.5 Bacterial Dysbiosis Index

In a study on human gut bacteria, changes in the abundance of bacteria associated with Crohn's disease, an inflammatory bowel disease, led to the identification of two bacterial taxa with strong co‐exclusion, which were used to calculate a bacterial dysbiosis index (BD) (Gevers et al. 2014). In the current study, we identified significant changes in gut bacterial abundance based on statistical analysis (*p* < 0.05), using the factor, larval quality (good or bad) in gilthead sea bream and European sea bass. After identifying taxa with significantly increased abundance in either good‐ or bad‐quality larval batches, a bacterial dysbiosis index was calculated as follows: BD = log [sum of the increase in the relative abundance of bacterial taxa in the gut microbiome of bad quality batches]/[sum of the increase in the relative abundance of bacterial taxa that were dominant in the gut microbiome of good quality batches]. The increase in abundance of each bacterial taxon in the gut was calculated by subtracting the average abundance of each taxon in the gut of good batches from the average abundance in the gut of bad batches, or by subtracting the average abundance in bad batches from the average abundance in good batches.

### 2.6 Detection of Amplicon Sequence Variants

Given the notable prevalence and potential pathogenicity of *Vibrio* spp. in the gut microbiota, we further characterised their amplicon sequence variants (ASVs). ASVs of 16S rRNA genes abundant in the gut from batches of larvae of bad quality tended to be pathogenic. A new universal primer set (UnipatVib F‐CAGTCGTGAGGAAGGKGGTRWK and UnipatVibR‐CACATCTGACTTAACKAACCACCTGC) was designed to amplify the 16S rRNA gene of putative pathogenic *Vibrio* spp. based on the workflow described in Najafpour et al. (2022). Briefly, the amplicons (168 bp) were cloned (pGEM‐T easy, Promega, USA) and used to transform *E. coli* (DH5α competent cells) and colony PCR products were sequenced to confirm primer specificity. Using the designed primer set, genomic extracts from the gut of larvae from good and bad quality batches were analysed in duplicate reactions by qPCR in a Bio‐Rad CFX96 instrument (Bio‐Rad Laboratories, Lisbon). The reaction volume was 10 μL and contained 200 nM of each primer, 80 ng of purified DNA (2 μL), or serial dilutions of the miniprep as the template (corresponding to 10^2^–10^7^ template copies per reaction) for the standard curve, 5 μL of 2× Forget‐Me‐Not EvaGreen qPCR Master Mix (Biotium) and 2.4 μL of sterile nuclease‐free water. Thermocycling conditions were 95°C for 2 min, followed by 40 cycles of 95°C for 5 s, 60°C for 10 s and 72°C for 10 s. A final melting curve was generated by increasing the temperature to 95°C in increments of 0.5°C every 10 s to confirm a single reaction product was obtained. Control reactions in which genomic DNA was substituted with water were used to confirm the absence of contamination in all qPCR experiments. The samples analysed by qPCR (*n* = 48) were those used for 16S rRNA gene sequencing plus additional larval gut samples collected from different quality production batches (*n* = 4 replicates/batch for site H1, H3, H4, H5, H6, and *n* = 6 replicates/batch for site H2).

## 3 Results

### 3.1 Sequencing Quality Control

A combined count of 4.7 million (M) Illumina 300‐bp reads was generated from 32 libraries (Table S2), corresponding to 3.2 M paired‐end reads (an average of 99,044 reads per library). Quality control and trimming procedures resulted in 2.4 million high‐quality reads (an average of 76,405 per library). The sequencing depth captured the diversity of the microbial community across most samples, as demonstrated by the alpha rarefaction curves that reached a plateau in 29 of the 32 libraries, signifying that most libraries achieved over 99% diversity coverage (Figure S1).

### 3.2 Bacterial Community Composition

**FIGURE 2:**
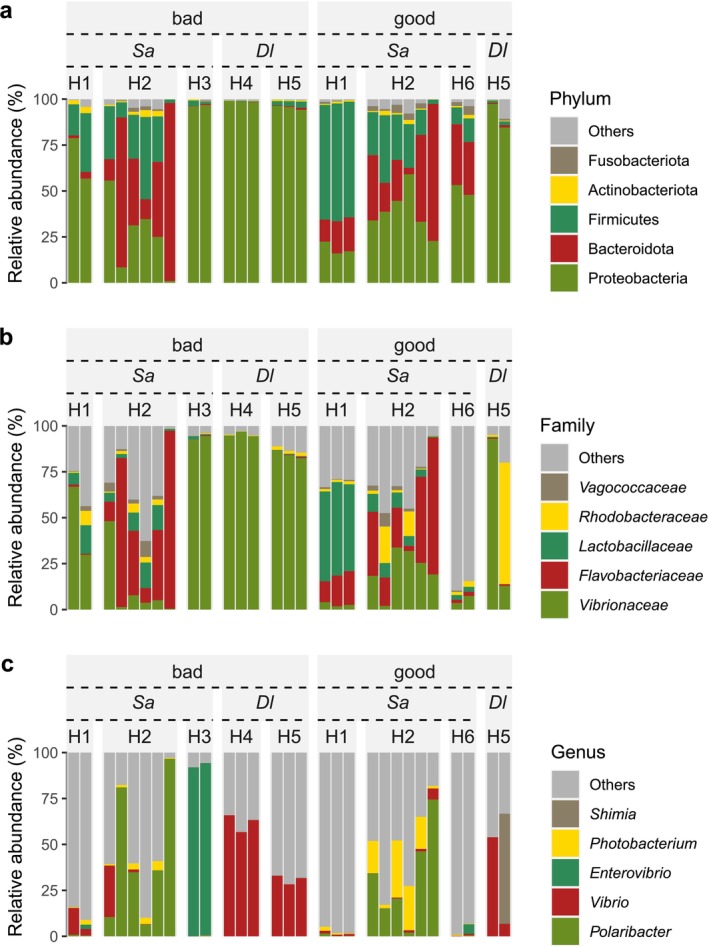
The dominant bacteria taxa at different taxonomic levels across all gut samples from good and bad larvae batches. ‘*Dl*’ represents European sea bass samples, and ‘*Sa*’ represents gilthead sea bream samples.

Gut microbial abundance according to taxonomy is listed in Table S2. The dominant phyla were Proteobacteria (bad batch mean abundance (*B*) = 66.5%; good batch mean abundance (*G*) = 44.2%), Firmicutes (*B* = 11.7%; *G* = 29.8%), Bacteroidota (*B* = 17.6%; *G* = 21.4%), Actinobacteriota (*B* = 1.5%; *G* = 1.4%) and Fusobacteriota (*B* = 0.1%; *G* = 1.4%) (Figure 2, Table S3). The most abundant families were *Vibrionaceae* (*B* = 55.6%; *G* = 19.7%), *Flavobacteriaceae* (*B* = 17.0%; *G* = 16.4%), *Lactobacillaceae* (B = 3.6%; *G* = 18.9%), *Rhodobacteraceae* (*B* = 2.0%; *G* = 11%) and *Vagococcaceae* (*B* = 1.4%; *G* = 1.4%) (Figure 2, Table S3). The most abundant genera were *Polaribacter* (*B* = 16.7%; *G* = 15.0%), *Vibrio* (*B* = 20.5%; *G* = 5.8%), *Enterovibrio* (*B* = 11.8%; *G* = 0.4%), *Photobacterium* (*B* = 1.1%; *G* = 7.5%) and *Shimia* (*B* = 0.0%, *G* = 4.5%) (Figure 2, Table S3 and Figure S2).

### 3.3 Alpha Diversity

**FIGURE 3:**
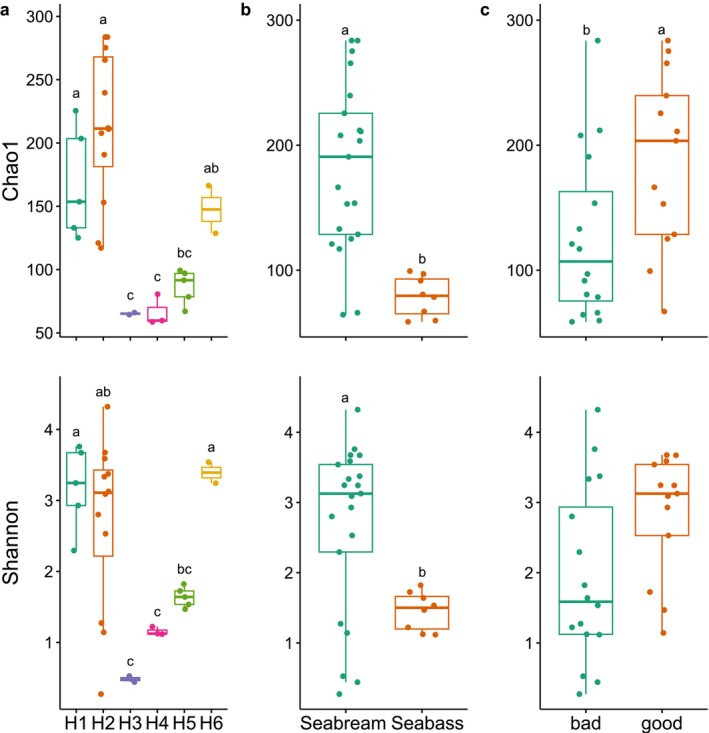
The box plots of alpha‐diversity indices (Chao1 and Shannon) based on site (a), species (b) and larval quality (c). Different letters represent statistically significant differences.

There were significant differences (*p* < 0.05) in bacterial species abundance and mean alpha diversity of the gut microbiome between hatchery sites (H1–H6), fish species (seabream and seabass) and larval quality (good and bad) (Figure 3 and Table S4). The Chao1 and Shannon alpha‐diversity indices differed significantly between the three factors (*p* < 0.05), except for the Shannon index and larval quality (Figure 3, Table S4). The alpha diversity in the seabass gut microbiome was significantly lower than the gut microbiome in seabream (*p* < 0.05). Alpha diversity was highest in site H2 and lowest in H3, H4 and H5, which included mainly bad larval batches. The GLMM confirmed a statistically significant relationship between Chao1 and larvae quality as a fixed effect and intercept (hatchery and fish species) in the model (Table 1). The estimate of good larval quality was significantly higher than zero (0.19), suggesting a positive relationship between good quality and Chao1 diversity (Table 1). Hatchery site had a larger contribution to the total variance in Chao1 (with hatchery and fish species as random effects) and a residual variance, when compared to the fish species, of approximately 50.3% (Table 1). However, the 95% confidence interval indicated uncertainty in the analysis, which may be associated with the small sample size. The mean effect of hatcheries on Chao1 reflected the influence of different hatcheries, H2 (0.62), H1 (0.37), H6 (0.16), H5 (−0.13), H3 (−0.24) and H4 (−0.26), while the mean effects of species included the effects of seabream (0.001) and seabass (−0.0008). The fitted GLMM did not identify a significant relationship between fixed effect (quality) and intercept (hatchery and fish species) for the Shannon diversity index.

**TABLE 1:** The output of the generalised linear mixed model fit by maximum likelihood for Chao1.

Random effects			
Groups	Name	Variance	Std. dev.
Hatchery	(Intercept)	0.075612	0.27498
Species	(Intercept)	0.000201	0.01417
Residual		0.074633	0.27319
Number of obs: 29, groups: Hatchery, 6; Species, 2			

Fixed effects				
	Estimate	Std. error	*t*‐value	Pr(> |*z*|)
(Intercept)	4.554616	0.007803	583.68	< 2e−16***
Good quality	0.195983	0.007781	25.19	< 2e−16***

*Note:* Signif. codes: 0 ‘***’.

### 3.4 Beta Diversity

**FIGURE 4:**
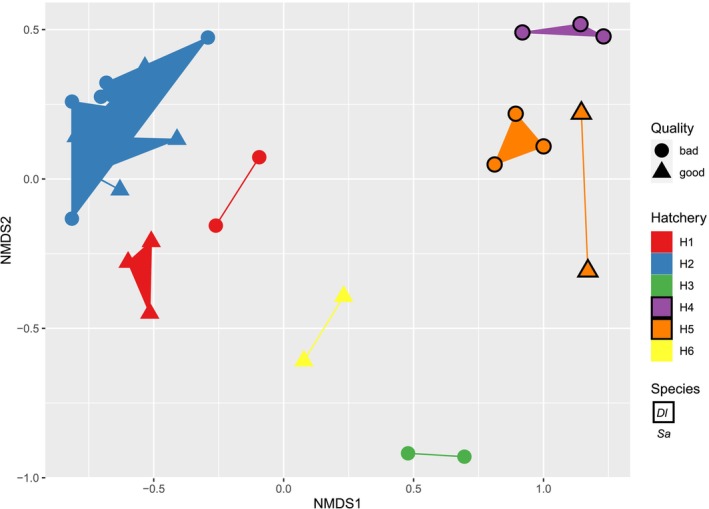
Nonmetric multidimensional scaling (NMDS) analysis of bacteria profiles of the gut samples. The samples include good and poor quality larval batches of gilthead sea bream (*Sa*) and European sea bass (Dl) from different hatcheries (H1–H6). Bray–Curtis distance was applied for this analysis in the phyloseq package, version 1.38.0.

The NMDS (Figure 4, Figure S3) and PERMANOVA (Table 2) both indicated that the gut microbial composition varied with hatchery site, species and larval quality. In H1 and H5, but not in H2, there was a noticeable separation of the gut microbial composition between good and bad quality larvae (Figure 4).

**TABLE 2:** PERMANOVA analysis of beta diversity differences based on quality (good and bad), hatchery (H1–H6) and species (gilthead sea bream and European sea bass) factors (permutations = 1000).

	df	SumOfSqs	*R* ^2^	*F*	Pr(> *F*)
Quality	1	0.8169	0.08257	5.1111	0.001***
Species	1	2.0482	0.20703	12.8157	0.001***
Hatchery	4	3.5122	0.35501	5.494	0.001***
Residual	22	3.516	0.3554		
Total	28	9.8933	1		

*Note:* Signif. codes: 0 ‘***’.

**FIGURE 5:**
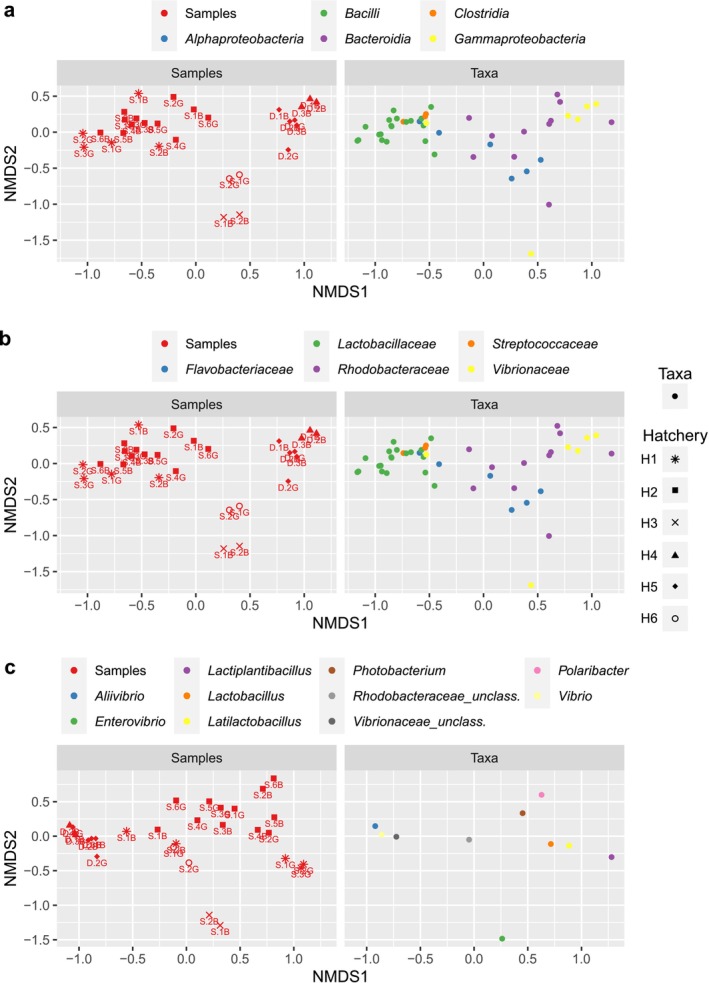
Split plots of nonmetric multidimensional scaling (NMDS) analysis of bacteria profiles of the gut samples. The samples and their associated abundant bacterial taxa are presented on two side‐by‐side panels. The graphs are organised by taxonomic level: Class (a), family (b) and genus (c). The ‘G’ in sample codes (e.g., S.2G) indicates good quality larvae, while ‘B’ (e.g., S.1B) indicates poor quality larvae.

Taxa abundance influenced sample clustering and the high relative abundance of *Vibrio* spp. and an unknown genus (Proteobacteria, *Vibrionaceae*) had a large contribution to the clustering of primarily the European sea bass gut samples from larvae categorised as bad quality (Figure 5). In line with this, the simper output confirmed a significant contribution of OTUs from *Vibrionaceae* to beta‐diversity in gilthead sea bream and European sea bass (Table S5).

The genera *Photobacterium*, *Latilactobacillus* and *Leucothrix* emerged as significant contributors to the disparity in the beta‐diversity of the gut microbiome between good and bad quality larvae, as indicated by the simper analysis output (Table S5). The high relative abundance of *Lactobacillus* and *Latilactobacillus* (Firmicutes, *Lactobacillaceae*) contributed to the clustering of gut samples from the good quality batches of gilthead sea bream larvae in H1 (Figure 5) and *Polaribacter* (Bacteroidota, *Flavobacteriaceae*) and *Photobacterium* (Proteobacteria, *Vibrionaceae*) were the main contributors to the clustering of gilthead sea bream gut samples from H2 (Figure 5).

### 3.5 Microbiome and Larval Quality

**FIGURE 6:**
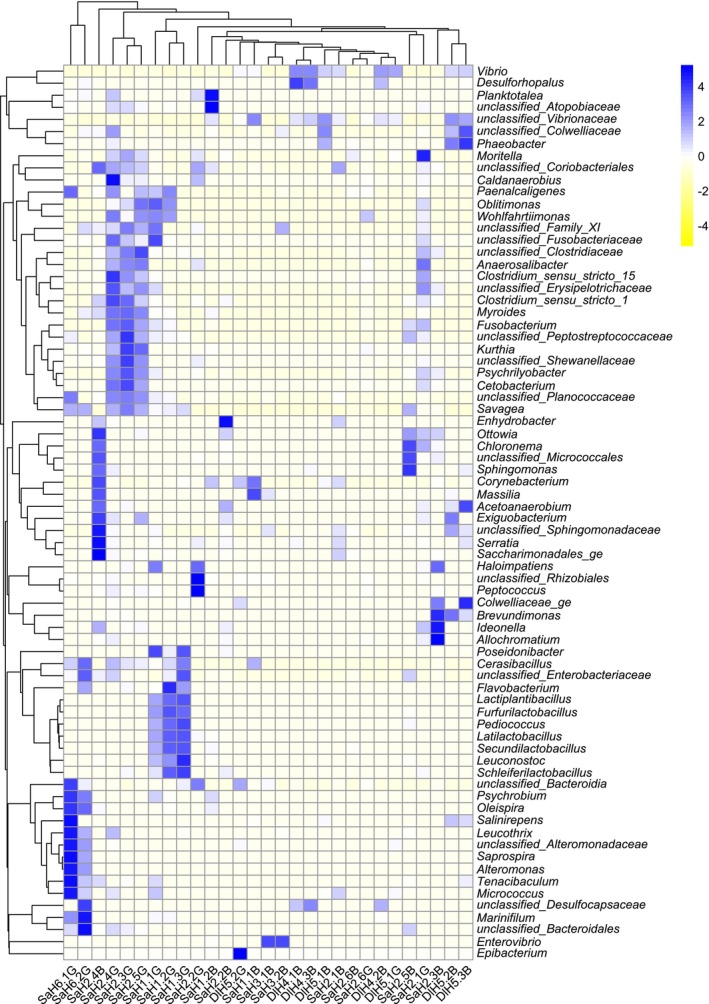
The heatmap of 74 OTUs with significant changes in their abundance based on larval quality (good and bad), determined by at least one statistical method (GLM, EdgeR and DESeq2). The values of these changes and their statistical confidence are detailed in Table S6.

Using multiple statistical methods at various taxonomic levels, a significant association between larval quality and bacterial abundance in the gut was identified (Table S6). Bacterial genera that significantly changed in abundance in the microbiome of the gut of good and bad quality larval batches, based on at least one statistical method, are presented in a heatmap (Figure 6).

The mean effect of hatchery and fish species, as random effects, on bacterial abundance at various taxonomic levels in the gut of larvae was estimated, with larval quality (good and bad) considered a fixed effect and bacterial markers associated with quality identified in GLM (Table S7). The gut microbiome of good larvae had a higher abundance of the Fusobacteriota taxa (bad batch mean (*B*) = 0.12%; good batch mean (*G*) = 1.4%), class of *Thermoanaerobacteria* (*B* = 0.0%; *G* = 0.1%) and family of *Moritellaceae* (*B* = 0.02%; *G* = 0.3%), *Shewanellaceae* (*B* = 0.1%; *G* = 1.3%), *Thiotrichaceae* (*B* = 0.2%; *G* = 1.8%) and *Clostridiaceae* (*B* = 0.4%; *G* = 1.4%) and genus of *Clostridium_sensu_stricto_15* (*B* = 0.0%; *G* = 0.07%), *Shewanellaceae_unclassified* (*B* = 0.0%; *G* = 0.06%), *Cetobacterium* (*B* = 0.0%; *G* = 0.24%), *Wohlfahrtiimonas* (*B* = 0.0%; *G* = 0.02%), *Psychrilyobacter* (*B* = 0.05%; *G* = 54%), *Moritella* (*B* = 0.0%; *G* = 0.3%), *Latilactobacillus* (*B* = 0.17%; *G* = 3.4%) and *Anaerosalibacter* (*B* = 0.0%; *G* = 0.06%). In contrast, the gut microbiome of bad quality larvae batches had a higher abundance of the bacteria class *Gammaproteobacteria* (*B* = 65.5%; *G* = 35%) and *Desulfobulbia* (*B* = 0.3%; *G* = 0.1%), family of *Vibrionaceae* (*B* = 56.5%; *G* = 21.6%), *Sphingomonadaceae* (*B* = 0.2%; *G* = 0.03%) and *Oxalobacteraceae* (*B* = 0.2%; *G* = 0.0%) and genus of *Vibrio* (*B* = 20.8%, *G* = 6.7%) and *Massilia* (*B* = 0.2%; *G* = 0.0%). In addition, the abundance of some bacterial taxa in the gut microbiome differed significantly between gilthead sea bream and European sea bass (Tables S5 and S6).

The BD index was calculated as the log [sum of the increase in the relative abundance of bacterial taxa of Proteobacteria, *Gammaproteobacteria*, *Desulfobulbia*, *Vibrionaceae* and *Sphingomonadaceae* dominant in the gut microbiome of bad quality batches]/[sum of the increase in the relative abundance of bacterial taxa of Fusobacteriota, Firmicutes, *Fusobacteriia*, *Thermoanaerobacteria*, *Moritellaceae*, *Shewanellaceae*, *Thiotrichaceae* and *Clostridiaceae* that were dominant in the gut microbiome of good quality batches]. The BD index was 0.72 when all taxa (phylum, class and family) were incorporated in the analysis of all hatcheries and species. The BD index for the individual hatcheries H1, H2 and H5, each containing good and bad quality larvae batches, was 0.42, 0.004 and 2.6, respectively.

### 3.6 The Association of Pathogens With Larval Quality and Species

**FIGURE 7:**
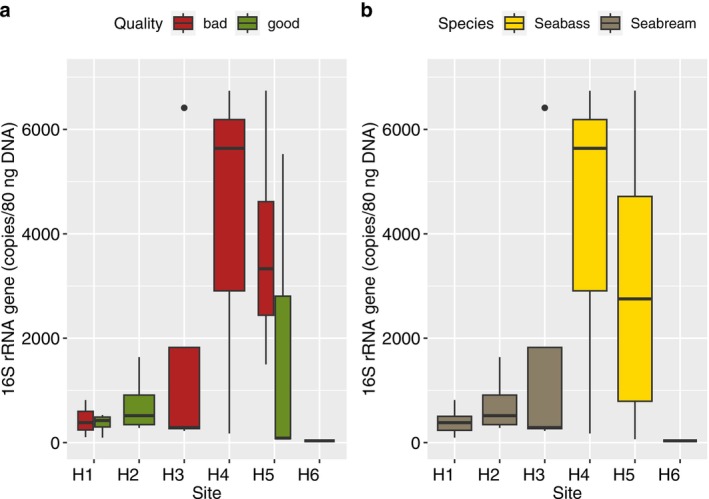
The average copy number of 16S rRNA gene of potential pathogenic *Vibrio* species amplified by quantitative PCR (qPCR) using a group‐specific primer set.

Analysis of 16S rRNA gene sequences generated by PCR of gut samples used for microbiome analysis and additional gut samples of good and bad quality larvae against the NCBI database confirmed the precise targeting by the designed primers of a group of pathogenic *Vibrio*. The sequences retrieved from NCBI that shared the highest sequence similarity (98.80%–100% identity) with the PCR amplicons of 16S rRNA gene sequences were *Vibrio alginolyticus* , *V. aestuarianus* , *V. cortegadensis* and *Vibrio* sp. Using the *Vibrio‐specific* quantitative PCR (qPCR), the average copy number amplified was higher in the gut microbiome of bad quality batches of sea bass larvae from H4 (Figure 7). The lowest copy number was obtained for the gut microbiome from a good quality batch of larvae from H6 (Figure 7). There was a significant difference in *Vibrio* copy number between sea bass and sea bream, with pairwise comparisons highlighting significant differences in copy number between the gut of bad quality batches of sea bass and both the good and bad quality batches of sea bream.

### 3.7 Correlation Network Between Bacteria Profiles

**FIGURE 8:**
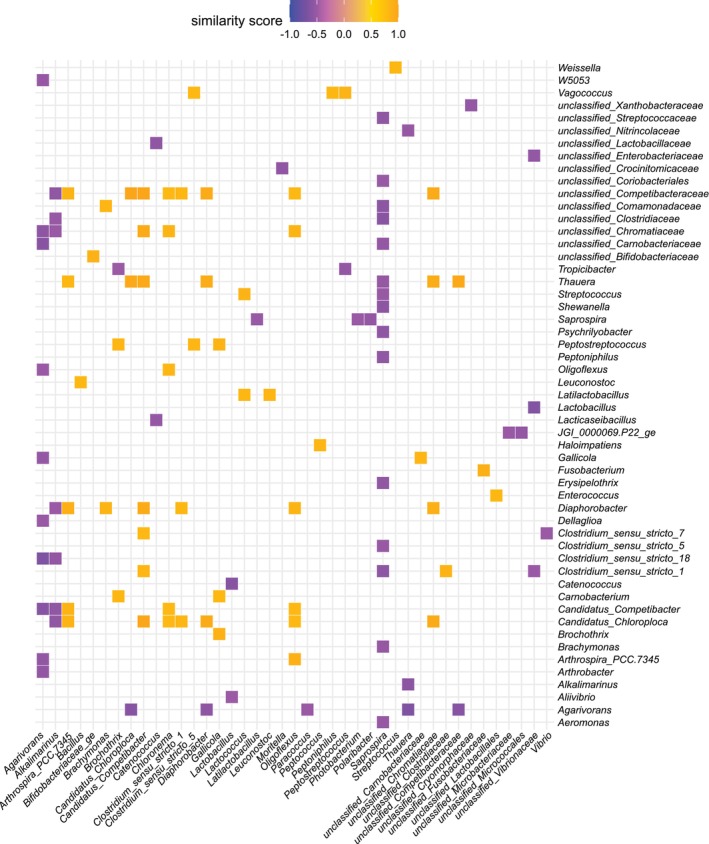
A subset of top association patterns between gut bacteria in gilthead sea bream and European sea bass. This correlation analysis included both good and bad‐quality larval batches.

A matrix of positive and negative similarity scores (*q*‐values < 0.1) was constructed for the gut bacterial associations from gilthead sea bream and European sea bass (Figure 8). The bacteria with the highest association scores, *Saprospira*, *Agarivorans* and *Alkalimarinus* had negative similarity scores with high numbers of bacterial genera (Figure 8). In contrast, *Candidatus Chloroploca*, *Candidatus Competibacter*, *unclassified Competibacteraceae* and *Diaphorobacter* had positive similarity scores with a higher number of bacterial genera and negative similarity scores with *Alkalimarinus* or *Agarivorans* (Figure 8).

**FIGURE 9:**
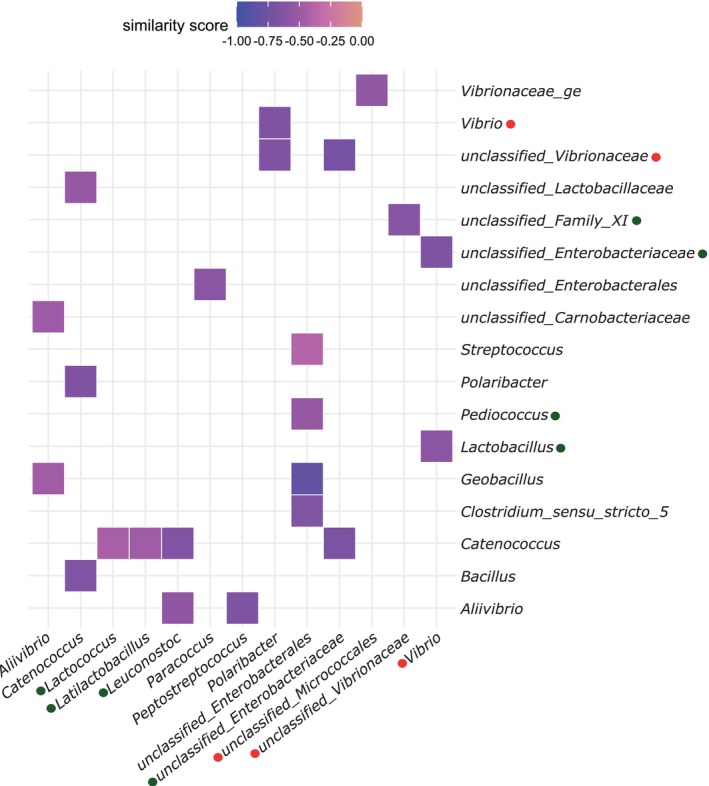
A subset of the top association patterns between gut bacteria by comparing good and bad quality larval batches. The green and red circles mark bacterial genera with high abundance in good and bad quality larval batches, respectively, and is based on the differential abundance analysis presented in Table S6.

A similarity matrix was constructed based on negative similarity scores (*q*‐value < 0.1) for the comparison between the gut microbiome of good and bad quality larvae batches (Figure 9). A negative similarity score between potential pathogens from the *Vibrionaceae* family (e.g., *Vibrio* spp.) and beneficial bacteria (e.g., *Lactobacillus*) was observed (Figure 9). There was a significant negative similarity score between the unclassified genus of *Vibrionaceae*, which was abundant in the gut of bad quality larvae batches, and *Family XI*, which was abundant in the gut of good quality larvae batches (Figure 9). A negative similarity score of *Catenococcus* (*Vibrionaceae*) with four abundant bacteria in good larvae batches, including *Lactococcus*, *Latilactobacillus*, *Leuconostoc* and an *unclassified‐Enterobacteriaceae*, was also detected.

### 3.8 Functional Prediction

**FIGURE 10:**
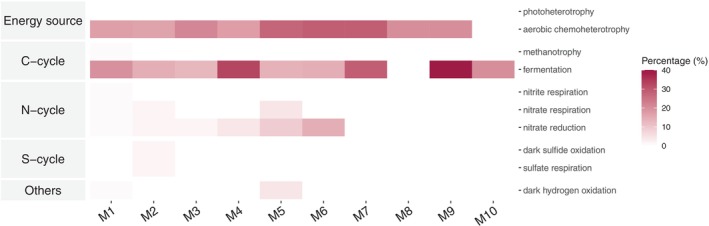
The functional analysis of predicting OTU percentages for each trait within a network module. This prediction used bacteria profiles of 29 gut samples from gilthead sea bream and European seabass (Table S8).

For the gut microbiome, the traits ‘aerobic chemoheterotrophy’ (mean ± SD, 23.8% ± 4.3%) and ‘fermentation’ (23.4% ± 3.9%) emerged as the most prevalent, closely followed by ‘nitrate reduction’ (2.9% ± 1.1%), ‘animal parasites or symbionts’ (2.5% ± 1.5%), and ‘aromatic compound degradation’ (1.1% ± 0.6%, Figure 10, Table S8). No significant correlation was found between the larval quality and the functional predictions.

## 4 Discussion

This study profiled the gut bacteria of gilthead sea bream and European sea bass larvae from commercial hatcheries, examining how hatchery, species and larval quality contributed to differences in gut bacterial richness and composition. Significant changes in the abundance of specific bacterial taxa linked to larval quality are proposed as markers of gut dysbiosis. The presence and abundance of pathogens, such as *Vibrio* spp. in the gut of larval batches classified as poor quality, highlight the importance of a balanced microbiota and the connection between dysbiosis and larval health, though it remains unclear if reduced diversity and increased pathogen abundance are causes or consequences of poor larval quality. The findings underscore the need for hatchery management strategies that promote a balanced and diverse gut microbiome. The observed negative correlation between certain gut bacteria suggests potential antagonistic interactions, and the correlation network of gut bacterial profiles could serve to inform therapeutic interventions to improve gut health. Overall, the gut microbial profiles of larvae from several different hatcheries, species and larval batches offer a foundation for developing robust approaches to enhancing larval fish gut health.

### 4.1 Dominant Gut Bacteria as Indicators of Larval Quality and Performance

Recent studies suggest that the signature of gut microbiota in fish can serve as an indicator of health, such as the correlation between a decrease in Fusobacteria and Firmicutes, alongside an increase in Proteobacteria (renamed *Pseudomonadota* in recent taxonomic revisions), and a diseased state (Mougin and Joyce 2023). However, contradictory results from studies on zebrafish and salmon have limited the development of this field. For example, one study on zebrafish showed a positive correlation between *Mycoplasma* spp. and parasite burden in the gut of animals infected with *Pseudocapillaria tomentosa*. Other studies proposed a mutualistic relationship between *Mycoplasma* spp. and their salmonid hosts and considered them to be part of the core microbiota (Llewellyn et al. 2016; Rasmussen et al. 2021). In contrast, *Mycoplasma* appeared only occasionally in Atlantic salmon and was not considered to be part of the core microbiome, even when fish were analysed from the same farm at different times, and despite the proposed beneficial effects, was associated with the spread of infections (Zarkasi et al. 2014; Parshukov et al. 2019).

In addition to the conflicting and variable results about the association between microbiota and gut health, identifying a taxonomic biomarker of gut health in fish can be challenging due to factors such as pathogen diversity and species‐specific host responses (Egerton et al. 2018). The divergent findings in relation to the microbiome in fish make the identification of a single biomarker (genus, taxa or species) that can be applied across a wide range of conditions or fish species extremely difficult (Mougin and Joyce 2023). The advantage of the present study lies in the use of two marine species from multiple commercial hatcheries, which were reared under different conditions, and this permitted the identification of conserved bacterial markers that were correlated with quality irrespective of species or production regime. A group of core and species/site‐specific bacterial markers were identified and a bacterial dysbiosis index (BDI) was proposed, which is a novel approach for fish. A range of bacterial taxa were incorporated into the BDI rather than focusing on a single taxa. The rationale for the use of multiple taxa was the reduced risk of false positive or negative results and since BDI was established using multiple species and hatchery conditions it is likely to be applicable across a broad range of conditions. The dominant phyla in the gut samples comprised Proteobacteria, Firmicutes, Bacteroidota, Actinobacteriota and Fusobacteriota. This is similar to the most abundant phyla observed in whole larvae of gilthead sea bream and European sea bass across multiple hatcheries, with Proteobacteria (mean = 59.6%) as the most abundant, followed by Bacteroidetes (mean = 12.2%), Firmicutes (mean = 3.4%) and Actinobacteria (mean = 0.8%) (Najafpour et al. 2023). The prevalence of Proteobacteria and Firmicutes has been reported in the gut microbiome of various fish species, including ray‐finned and cartilaginous fish obtained from seawater and freshwater environments (Kim et al. 2021). Considering our results in the gut of larval gilthead sea bream and European sea bass, and the presence of Proteobacteria and Firmicutes in whole larvae at flexion and mid‐metamorphosis (Najafpour et al. 2023), as well as reports about the gut microbiome in several fish species (Kim et al. 2021), we hypothesise that Proteobacteria and Firmicutes are part of the core gut microbiome in teleost fish. However, the actual contribution of these phyla to the host requires further experimental validation and could be improved by the use of well‐developed models, such as Bayesian network models (Moroni et al. 2025). Overall, the predicted high contribution of these two phyla to the fish gut microbiota aligns with findings reviewed by Ghanbari et al. (2015), who reported that approximately 90% of fish intestinal microbiota are composed of Proteobacteria, Bacteroidetes and Firmicutes.

Proteobacteria occurred in the gut of bad and good quality sea bream and sea bass larvae batches. An elevated average abundance of Proteobacteria, lower average abundance of Firmicutes and Bacteroidetes and reduced microbial diversity occurred in the bad‐quality batches, which could indicate gut microbiome dysbiosis. An overall rise in the ratio of Firmicutes/Bacteroidetes was a characteristic of good‐quality larval batches across different hatcheries and larval batches. However, the application of this concept in fish requires further investigation to determine the robustness or limitations of using this ratio as a biomarker of fish gut health and quality in aquaculture. In humans, although Firmicutes and Bacteroidetes are also the predominant gut phyla (Eckburg 2005; Qin et al. 2010), a higher Firmicutes/Bacteroidetes ratio has been associated with obesity (Magne et al. 2020). However, many studies on humans lack the statistical power needed to detect modest differences between healthy and obese individuals, suggesting that this ratio may not be a reliable marker of microbiome dysbiosis in the context of obesity (Eckburg 2005; Qin et al. 2010; Magne et al. 2020). While Fusobacteria might not be as prevalent as Firmicutes/Bacteroidetes in marine fish (Kim et al. 2021), our findings suggest they may mitigate dysbiosis in the gut and be associated with good larval quality. Other studies support this idea. For example, in adult grass carp ( *Ctenopharyngodon idella* ), the application of antibiotics, which disrupt the gut microbiota, triggered an expansion of Proteobacteria and the concurrent suppression of Fusobacteria and negatively affected the expression of epithelial cell tight junction and inflammation‐related genes (Sun et al. 2021). Similarly, in adult common carp ( *Cyprinus carpio* ) infected with spring viremia of carp virus (SVCV), there was a marked increase in the abundance of Proteobacteria and a decrease in Fusobacteria (Meng et al. 2021). A reduction in gut microbial diversity and structure was also associated with an increased abundance of *Vibrio* and a decreased abundance of Bacteroides and *Fusobacterium* in starved adult grass carp (Tran et al. 2018; Luan et al. 2023).

At the class level, a higher abundance of *Fusobacteriia* and *Thermoanaerobacteria* in the gut of good quality larval batches and an abundance of *Gammaproteobacteria* and *Desulfobulbia* in bad quality batches indicate they may be potential biomarkers of larval quality. It should be noted that these classes were more abundant in the gut of good quality larval batches mainly from hatchery H2, indicating that it is not part of the conserved core bacteria in the larval fish gut. *Thermoanaerobacteria* have a preference for higher ambient temperatures (Harnvoravongchai et al. 2020), and differing water temperature in hatcheries may explain this result although due to limited access to commercial hatchery records it was unclear if this was the case for H2. The higher abundance of *Desulfobulbia* in bad quality European seabass larvae batches, combined with its absence in most other samples, indicates that it could serve as a larval quality biomarker and that this class is not a part of the core gut bacterial community. These findings align with research in humans, where sulphate‐reducing prokaryotes are not numerically dominant in the gut microbiome, although the sulphide produced by these bacteria was linked to adverse effects on gut health, including the development of inflammatory bowel disease (IBD) and gut dysbiosis (Rabus et al. 2015; Ijssennagger et al. 2016; Sultan et al. 2021). Similarly, based on the output of the current study, the presence of *Gammaproteobacteria* is suggested as putative indicators of bad quality larvae due to their higher abundance in such larvae and since this genus contains potential pathogens for both fish and humans such as *Vibrio* and *Pseudomonas* (Williams et al. 2010; Sanches‐Fernandes et al. 2022; Ziarati et al. 2022).

At the family level, *Vibrionaceae* tended to dominate in the gut of larvae from bad quality batches. While the *Vibrionaceae* contain pathogenic and non‐pathogenic bacterial species, their tendency to dominate in the gut of bad quality larval batches may also lead to an increase in the abundance of pathogenic members. This was supported by quantitative measurements of specific pathogenic species using qPCR in European sea bass batches. The higher abundance of *Moritellaceae*, *Shewanellaceae*, *Thiotrichaceae* and *Clostridiaceae* in the gut microbiome of good quality larval batches suggests they may have beneficial effects on quality. This observation is in line with the use of *Clostridium* and *Shewanella* as probiotics, and the reported symbiotic relationship between *Thiotrichaceae* and aquatic organisms (Chen et al. 2017; Cámara‐Ruiz et al. 2020; Guo et al. 2020). At the genus level, *Vibrio*, and to a lesser extent, *Massilia*, were associated with bad quality larval batches or gut dysbiosis. In a previous study of eggs and whole larvae from the same commercial hatcheries for European sea bass and gilthead sea bream, *Vibrio* was part of the core microbiome, while *Massilia* was not. The results suggest *Vibrio* abundance is a marker of poor quality larvae and dysbiosis in commercially produced gilthead sea bream and European sea bass, while *Massilia*, as occurs in humans, may be associated with pathogen‐dependent dysbiosis (Mizutani et al. 2021).

The potential beneficial relationship of species of *Clostridium_sensu_stricto_15*, *Shewanellaceae_unclassified*, *Cetobacterium*, *Psychrilyobacter*, *Moritella* and *Latilactobacillus* within the gut of fish larvae is suggested by the current study. *Lactobacillus* species are regarded as favourable microorganisms in the gut of finfish, and beneficial effects include promotion of host gastrointestinal development, digestive function, mucosal tolerance, immune responses and disease resistance (Ringø et al. 2018). Furthermore, *Cetobacterium* ( *C. somerae* ) in the gut contributes to the production of Vitamin B12, an essential vitamin for blood and nerves (Qi et al. 2023). *Secundilactobacillus*, recently reclassified from *Lactobacillus* (Das et al. 2022), had a higher abundance in the gut microbiome of good‐quality larvae batches in the present study.

In general, a bacteria genus can include both pathogenic and beneficial species. *Moritella viscosa* is a pathogen of winter ulcer disease, particularly in salmonid fish in seawater during colder periods, and causes skin lesions and ulcers (Løvoll et al. 2009; Ramberg et al. 2022). Interestingly, the elevated abundance of *Moritella* spp. in the gut microbiome of good‐quality batches of larval gilthead sea bream suggests some members of this genus may be beneficial. This might be explained, for example, by the production of beneficial factors such as docosahexaenoic acid (DHA), an omega‐3 fatty acid (Kautharapu and Jarboe 2012; Kautharapu et al. 2013). If *Moritella* contributed positively to gut health of larval gilthead sea bream and European sea bass remains to be investigated.

Antibiotic resistance poses significant risks to organisms, including fish and aquaculture species. Probiotics have been proposed as a measure to mitigate these risks and promote health in both humans and animals. In aquaculture, probiotics enhance health and growth of aquatic organisms by inhibiting the growth of harmful pathogens, improving disease resistance, aiding nutrient digestibility and increasing stress tolerance (Calcagnile et al. 2024). However, more research is needed about probiotics to address knowledge gaps, such as selecting and optimising probiotics for specific species, developing effective delivery methods and assessing their potential ecological risks (Amenyogbe 2023). To address some of these knowledge gaps, correlation analysis linking animal phenotype and gut bacteria may provide valuable insights into potential species‐specific or universal probiotics. For example, the negative correlation between some of the abundant bacterial genera in the gut microbiome of larvae of good quality compared to those of bad quality, such as *unclassified_Family_XI* (order *Peptostreptococcales‐Tissierellales*) and *unclassified_Enterobacteriaceae*, suggests they may be candidate enhancers of fish gut health. Furthermore, the strong negative correlations observed for bacterial genera such as *Saprospira*, *Agarivorans* and *Alkalimarinus* with numerous other taxa warrant further investigation as well as their potential antagonistic effects against pathogens.

### 4.2 The Risk of Pathogenic Species and Dysbiosis

Bacterial taxa at various taxonomic levels were identified as potential quality markers through differential abundance analysis. Using multiple quality markers provides a more robust and realistic index than using a single taxon when calculating bacterial dysbiosis in aquaculture due to the inherent variability of fish larvae and commercial production regimes. Dysbiosis has mostly been studied in mammals and is characterised by the loss of beneficial microbes, the proliferation of pathobionts and decreased diversity within the resident commensal community (Petersen and Round 2014). The BD index devised for humans and adjusted for aquaculture seems to predict bacterial dysbiosis of the gut microbiome under hatchery conditions, and a positive BD index above 0.4 in the gut of bad quality larvae was taken to be indicative of dysbiosis. Dysbiosis in the gut of poor‐quality European sea bass and gilthead sea bream larval batches is predictable in the context of decreased alpha‐diversity (e.g., Chao1) and increased abundance of genera containing pathogenic bacteria such as *Vibrio*. Consistent with the results of the present study, dysbiosis in other aquaculture species, such as shrimp, oysters and adult fish, revealed diminished bacterial alpha diversity and increased pathogenic bacteria abundance (Infante‐Villamil et al. 2021). Notably, the association found in the present study between the gut bacterial community and the size of gilthead sea bream and European sea bass larvae was also identified for the mangrove killifish, *Kryptolebias marmoratus* and Atlantic cod, *Gadus morhua* (Forberg et al. 2016; Trinh et al. 2017). However, the killifish and Atlantic cod studies of the microbiome used 16S rRNA PCR and denaturing gradient gel electrophoresis (DGGE), which do not capture the entire bacterial diversity compared to 16S rRNA high throughput gene sequencing. Therefore, the present study that determined the gut microbiome of multiple larval batches from commercial hatcherys using 16S rRNA gene sequencing linked larval performance and the gut bacterial community at a level not previously reported.

Further evaluation of the bacterial taxonomic units abundant in the gut of poor quality larval batches suggests that dysbiosis was linked to an increase in pathogenic bacteria. The dominance of *Vibrio* species in the gut microbiome of larvae from poor batches in the present study is a good example of this pattern, and their virulence in fish and humans, as well as their impact on other beneficial bacteria, has already been demonstrated (Baker‐Austin et al. 2018; Barrasso et al. 2022; Manchanayake et al. 2023). Specific pathogens, including *V. alginolyticus* , *V. aestuarianus* and *V. cortegadensis*, were identified in the gut microbiome of the gilthead sea bream and European sea bass in the present study. The mass mortalities in European sea bass and gilthead sea bream aquaculture caused by *V. alginolyticus* (Moustafa et al. 2015; Ragab et al. 2022) underscore the imperative of further investigation and the development of management tools to reduce the risk of pathogenic species. Furthermore, the presence of *V. aestuarianus* and *V. cortegadensis*, alongside *V. alginolyticus* , in hatchery production of gilthead sea bream and European sea bass, and their predominance in the gut microbiome of batches of poor quality larvae, particularly in the case of European sea bass further emphasises their potential contribution to dysbiosis. The average copy number of the targeted pathogens in the gut of larvae from poor quality production batches of European sea bass was > 2000, suggesting it may be possible to establish a threshold value linked to the quality of this species using the designed primer set.

### 4.3 The Impact of Site and Species on the Microbial Community of the Gut

The beta diversity suggests a distinct microbial community within the gut of gilthead sea bream and European sea bass and between hatchery sites. The influence of hatchery site on the microbial community was also observed in our previous studies of gilthead sea bream and European sea bass eggs and whole larvae (Najafpour et al. 2021, 2023). Furthermore, distinct species‐specific microbiomes were observed for the eggs of gilthead sea bream and European sea bass (Najafpour et al. 2021). Interestingly, in contrast to the species specificity of the gut microbiomes in the present study, for the whole larvae microbiomes the fish species was not a major factor driving beta‐diversity (Najafpour et al. 2023). In the current study, both hatchery site and fish species influenced the gut bacteria and this is most likely explained by differences in rearing practices, such as feeding regime, food additives and the environment. Variations in the ontogeny of gut development and function, including the hosts immune response to microbiota, presumably explain the significant differentiation between the gut microbiome of gilthead sea bream and European sea bass. However, since the different species studied were produced in different hatcheries, the relative impact of the environment on the gut microbiome could not be established.

## 5 Conclusion

The present study indicates differences in alpha diversity exist between the gut microbiome of European sea bass and gilthead sea bream larvae from different European hatcheries, with a tendency to increase in good quality production batches. Furthermore, the data suggested that larval quality, hatchery site and species influenced beta diversity. An illustrative example is the clustering of European sea bass samples into batches categorised as poor quality, primarily driven by *Vibrio* and an unknown genus (Proteobacteria, *Vibrionaceae*). In contrast, the clustering of good quality larvae of gilthead sea bream was driven by the high relative abundance of *Lactobacillus* and *Latilactobacillus* (Firmicutes, *Lactobacillaceae*). The study found a link between dysbiosis in gut microbiota and suboptimal larval performance. The average abundance of bacterial taxa related to good and bad quality larval batches can serve as markers for anticipating dysbiosis and initiating timely interventions to regulate the gut microbiota. This study introduced a novel approach for calculating the Bacterial Dysbiosis Index (BDI) that incorporates a broader spectrum of bacterial taxa, including Proteobacteria, *Gammaproteobacteria*, *Desulfobulbia*, *Vibrionaceae* and *Sphingomonadaceae* (dominant in poor‐quality batches) as well as *Fusobacteriota*, *Firmicutes*, *Fusobacteriia*, *Thermoanaerobacteria*, *Moritellaceae*, *Shewanellaceae*, *Thiotrichaceae* and *Clostridiaceae* (dominant in high‐quality batches). This taxonomically comprehensive approach offers a more robust measure of dysbiosis, with positive BDI values, particularly those above 0.4, serving as reliable indicators across various environmental conditions. The universal primers designed to amplify potential pathogenic *Vibrio* spp. identified in the OTUs of the gut microbiome of poor quality larvae could be used to assess dysbiosis, especially in European sea bass. Analysis of bacterial interactions suggested that the host species probably modulates the gut microbiota and determines the pathogenicity of specific bacterial species. Further studies are needed to analyse in greater detail the microbiota origin, interactions and influence on larval quality and to evaluate the attributes of beneficial bacteria of the gut microbiome. If the presence of pathogens like *V. aestuarianus* and *V. cortegadensis* primarily in the gut of European sea bass increases the risk of disease if dysbiosis occurs requires investigation. This study provides the foundation for the development of effective gut microbiota management strategies for European sea bass and gilthead sea bream hatcheries in the future.

## Author Contributions

**Babak Najafpour:** conceptualization, investigation, writing – original draft, methodology, validation, visualization, writing – review and editing, software, formal analysis, data curation. **Adelino V. M. Canario:** project administration, resources, supervision, writing – review and editing. **Deborah M. Power:** conceptualization, writing – review and editing, project administration, supervision, resources, validation, funding acquisition, methodology.

## Conflicts of Interest

The authors declare no conflicts of interest.

## Supporting information


**Table S1:** The metadata of gut samples of gilthead sea bream and European sea bass. The list and codes of samples used for DNA extraction and microbiome analysis, and the average weight and quality information for each sample are presented.

**Table S2:** Sequencing statistics of the 16S rRNA gene libraries of gut samples of gilthead sea bream and European sea bass. Three samples that produced a low number of reads were excluded from the analysis including estimations of alpha and beta diversity and statistical analysis. The sample information is provided in Table S1.

**Table S3:** The relative proportions of the detected bacteria at the level of the phylum, class, order, family and genus in the gut microbiome libraries obtained from gilthead sea bream and European sea bass larvae. The sample information is provided in Table S1.

**Table S4:** The output of statistical analysis to compare alpha‐diversity differences based on quality, species and hatchery factors.

**Table S5:** The most abundant and/or most variable OTUs that contributed to microbiome beta‐diversity based on quality, species and hatchery factors. The simper functions performed pairwise comparisons of groups of sampling units and identified the average contributions of each OTU to the average overall Bray–Curtis dissimilarity. SD = standard deviation of contribution, ratio = average to SD ratio, ava & avb = average abundances per group, *p* = permutation *p*‐value, cumsum = ordered cumulative contribution.

**Table S6:** Bacteria taxa (phylum, class, family and genus) with significantly different abundance based on the factors quality and species. Three statistical methods were applied and included Generalised Linear Model (GLM), EdgeR and DESeq2. In edgeR output, a positive log‐fold change indicates higher abundance in the good batch, while in DESeq2, a negative log‐fold change indicates higher abundance in the good batch.

**Table S7:** The mean effect of each specific hatchery and species on computed differential bacteria abundance at different taxonomic levels.

**Table S8:** The functional prediction of the percentages of the OTUs for each trait in network modules using gut bacteria profiles of gilthead sea bream and European sea bass larvae. The sample information is provided in Table S1.

**Figure S1:** Rarefaction plots of sequencing data from the 16S rRNA microbiome libraries related to European sea bass and gilthead sea bream gut samples collected in January 2018. The plot gives an indication of the bacterial diversity within the samples, determined using all operational taxonomic units (OTUs) found in each samples. The sample information is provided in Table S1.

**Figure S2:** Pie charts of the most abundant genera found in good (G) and bad (B) quality larval batches of European sea bass and gilthead sea bream.

**Figure S3:** The analysis of distance between microbial communities of gut samples of gilthead sea bream and European sea bass using different orientation methods. The sample information is provided in Table S1.

## Data Availability

The metagenomics raw data generated during this study were deposited at NCBI SRA (sequence read archive) under project number PRJNA1188567.
