# Supplementary material for: Bacterial 16S Ribosomal Gene Fingerprints as a Tool to Diagnose and Mitigate Fish Larvae Gut Dysbiosis
Source: Environ Microbiol Rep. 2025 Oct 3;17(5):e70187. doi: 10.1111/1758-2229.70187 (PMC12492350; doi:10.1111/1758-2229.70187)
Supplement: Supplementary file 9 — Figure S1: Rarefaction plots of sequencing data from the 16S rRNA microbiome libraries related to European sea bass and gilthead sea bream gut samples collected in January 2018. The plot gives an indication of the bacterial diversity within the samples, determined using all operational taxonomic units (OTUs) found in each samples. The sample information is provided in Table S1. [file EMI4-17-e70187-s003.pdf]

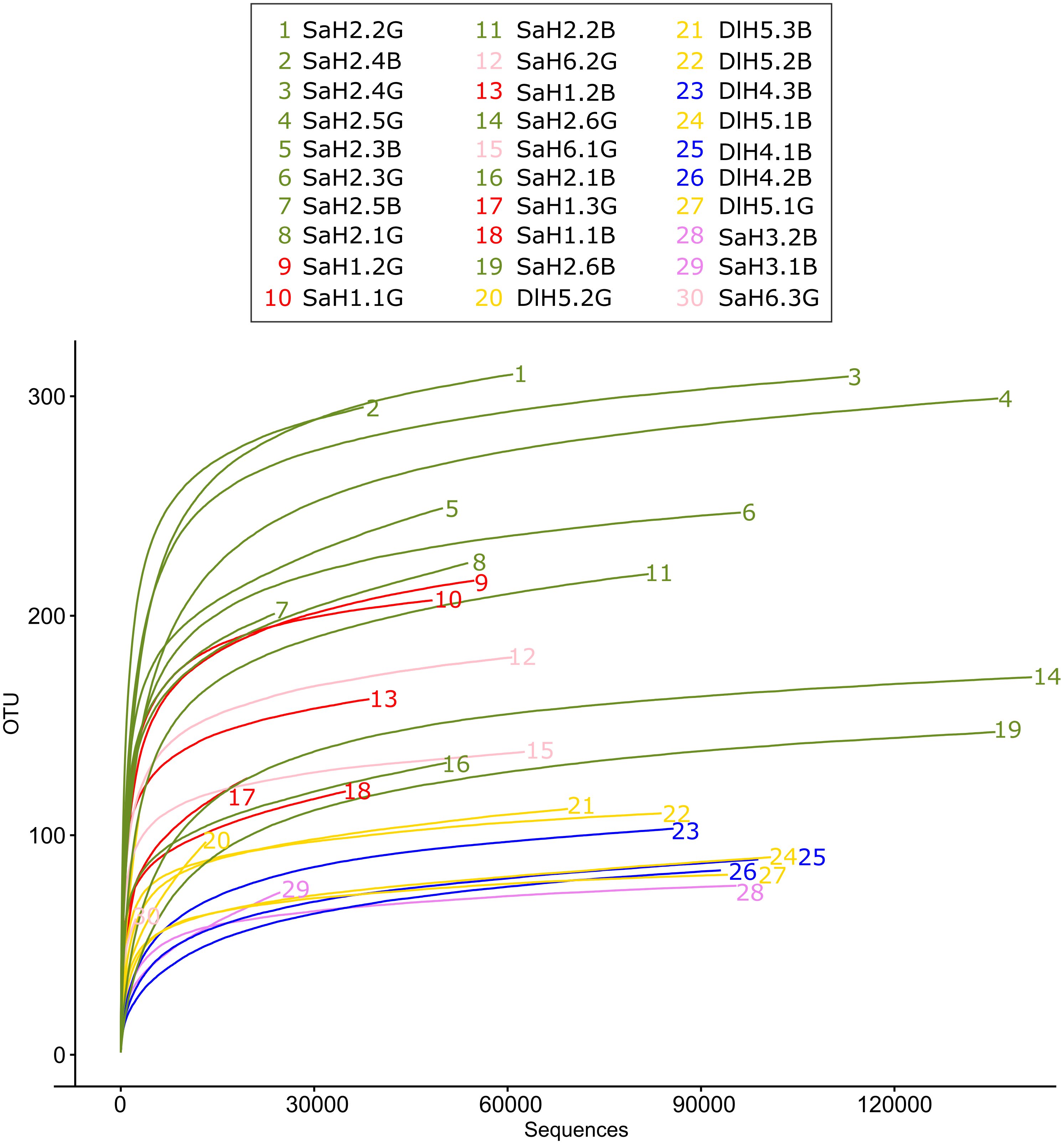

1 SaH2.2G
2 SaH2.4B
3 SaH2.4G
4 SaH2.5G
5 SaH2.3B
6 SaH2.3G
7 SaH2.5B
8 SaH2.1G
9 SaH1.2G
10 SaH1.1G
11 SaH2.2B
12 SaH6.2G
13 SaH1.2B
14 SaH2.6G
15 SaH6.1G
16 SaH2.1B
17 SaH1.3G
18 SaH1.1B
19 SaH2.6B
20 DIH5.2G
21 DIH5.3B
22 DIH5.2B
23 DIH4.3B
24 DIH5.1B
25 DIH4.1B
26 DIH4.2B
27 DIH5.1G
28 SaH3.2B
29 SaH3.1B
30 SaH6.3G
OTU
0
100
200
300
Sequences
0
30000
60000
90000
120000
