# Supplementary figures and images for: Bacterial 16S Ribosomal Gene Fingerprints as a Tool to Diagnose and Mitigate Fish Larvae Gut Dysbiosis
Source: Environ Microbiol Rep. 2025 Oct 3;17(5):e70187. doi: 10.1111/1758-2229.70187 (PMC12492350; doi:10.1111/1758-2229.70187)

B

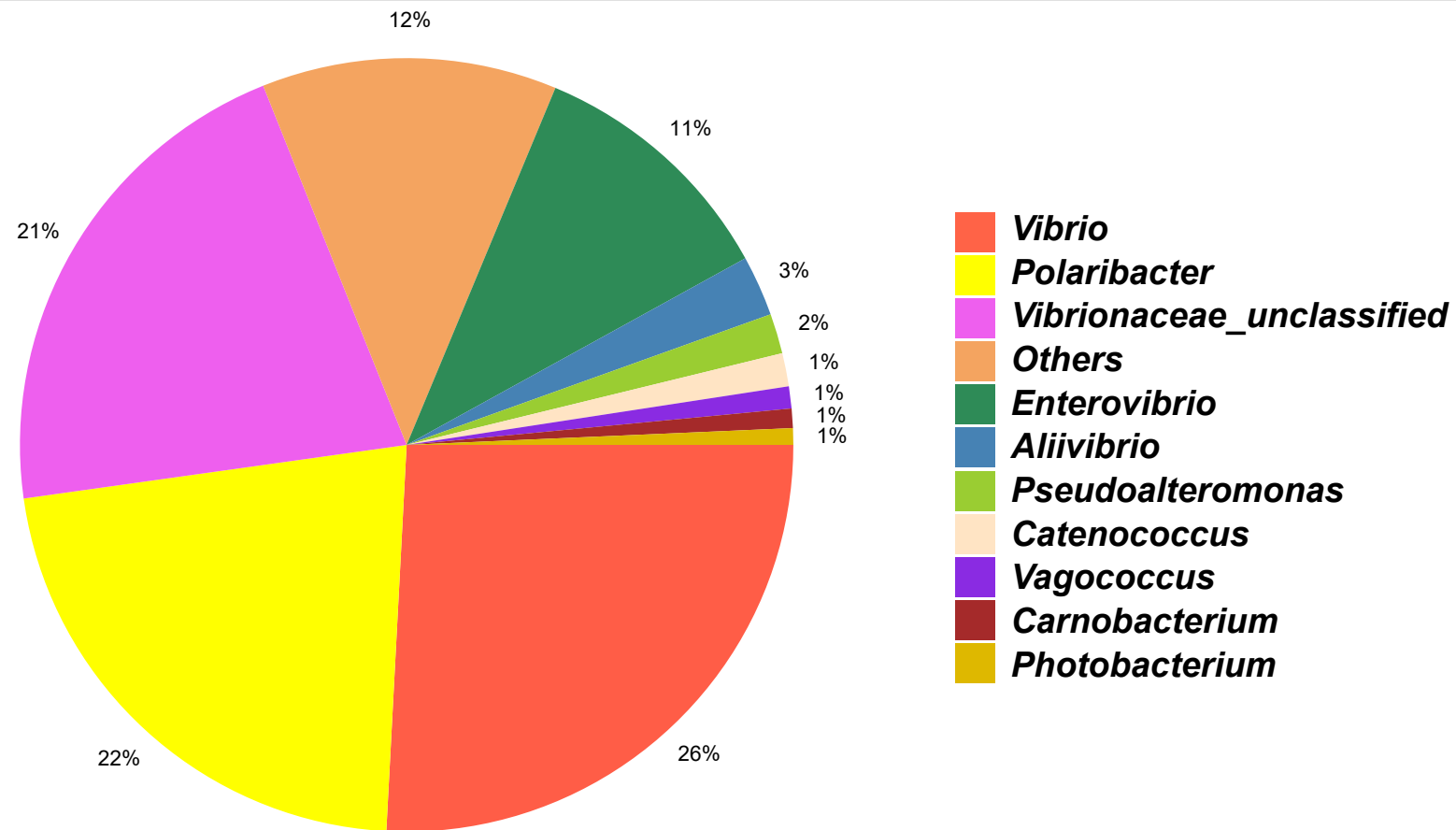

G

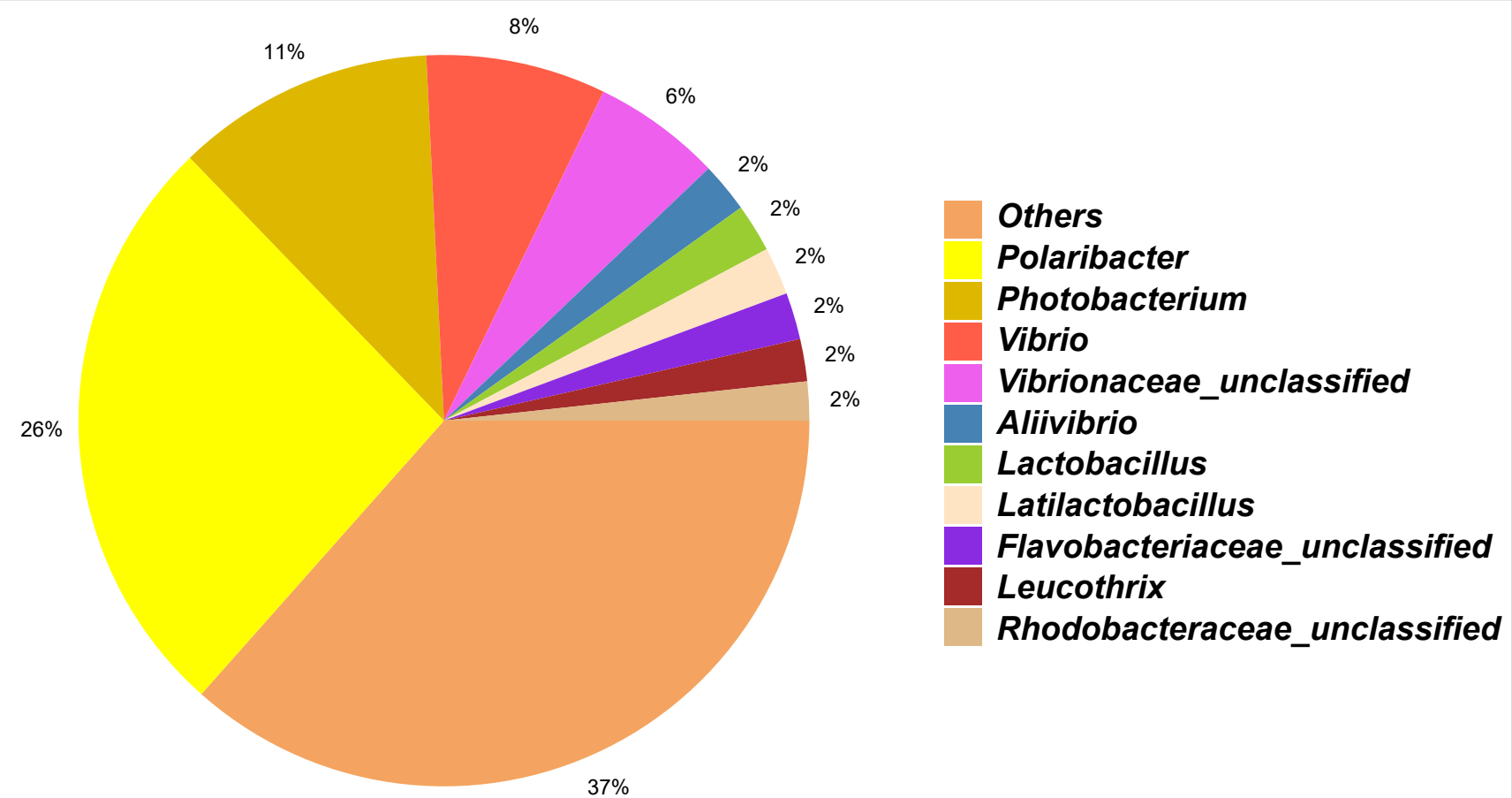

Supplement: Supplementary file 10 — Figure S2: Pie charts of the most abundant genera found in good (G) and bad (B) quality larval batches of European sea bass and gilthead sea bream. [file EMI4-17-e70187-s001.pdf]

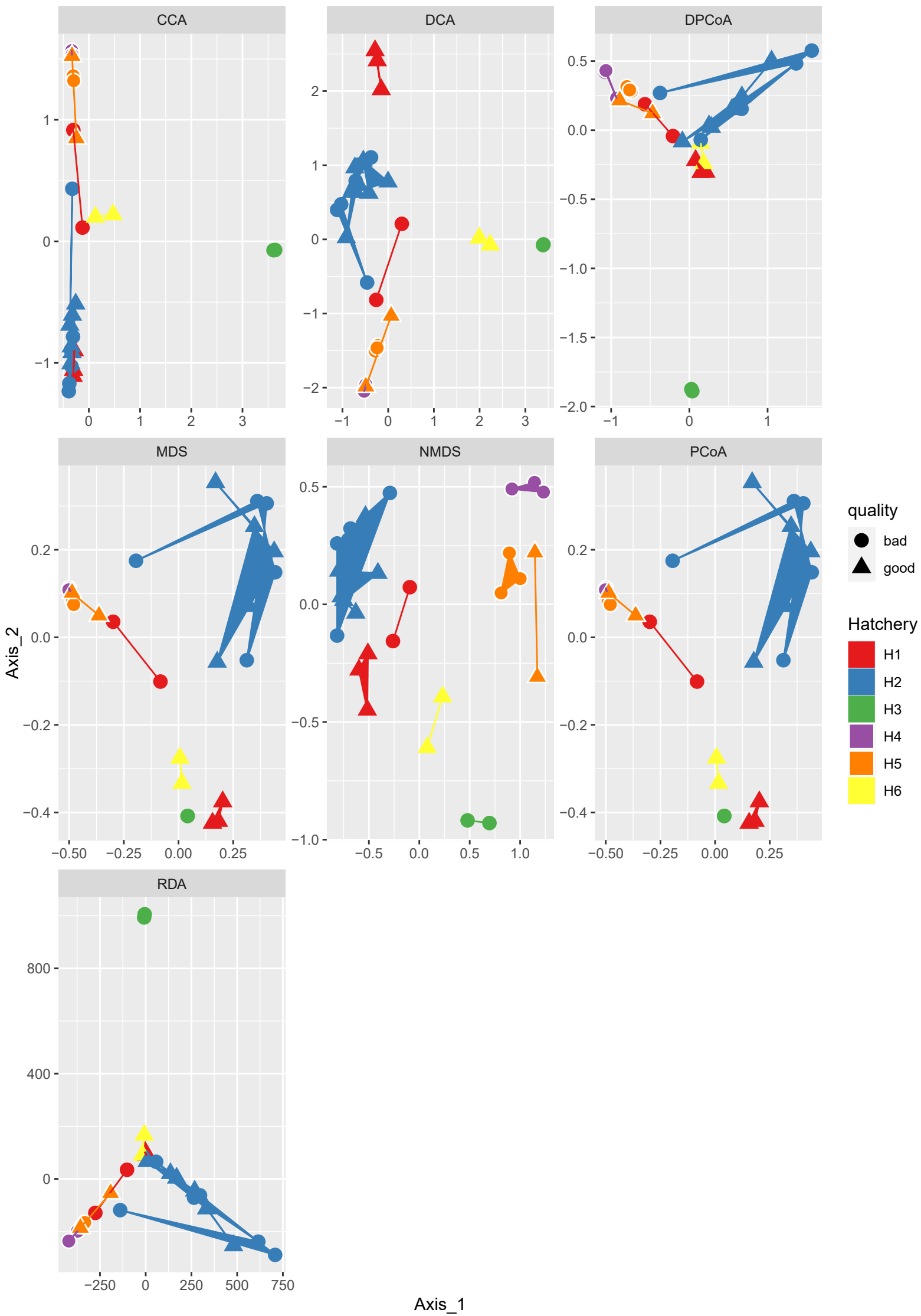

Supplement: Supplementary file 11 — Figure S3: The analysis of distance between microbial communities of gut samples of gilthead sea bream and European sea bass using different orientation methods. The sample information is provided in Table S1. [file EMI4-17-e70187-s008.pdf]
